# From Innovation to Implementation: The Evolution of HIV Pre-Exposure Prophylaxis and Future Implications

**DOI:** 10.3390/pathogens12070924

**Published:** 2023-07-09

**Authors:** Marta Rosas Cancio-Suárez, Jorge Díaz-Álvarez, Raquel Ron, Javier Martínez-Sanz, Sergio Serrano-Villar, Santiago Moreno, Matilde Sánchez-Conde

**Affiliations:** 1 Infectious Diseases Department, Hospital Ramón y Cajal, Carretera de Colmenar Km 9.1, 28034 Madrid, Spainjavier.jmas@gmail.com (J.M.-S.); serranovillar@gmail.com (S.S.-V.); smoreno.hrc@salud.madrid.org (S.M.); 2 Ramón y Cajal Research Institute (IRYCIS), 28034 Madrid, Spain; 3 CIBER de Enfermedades Infecciosas (CIBERINFEC), 28029 Madrid, Spain; 4 Department of Medicine, University of Alcalá de Henares, Guadalajara Campus, 28801 Alcalá de Henares, Spain

**Keywords:** pre-exposure prophylaxis, HIV, prevention strategies

## Abstract

Pre-exposure prophylaxis (PrEP) is a highly effective HIV-prevention strategy that involves the continuous administration of antiretroviral drugs to HIV-negative individuals with a substantial risk of contracting an HIV infection. The use of PrEP has shown a reduction in the risk of HIV acquisition through sexual intercourse by up to 99%. Despite its effectiveness, PrEP uptake remains low among populations at high risk of HIV infection. This highlights the need for further research in strategies to enhance awareness and uptake of PrEP amongst these specific populations. This article presents a comprehensive overview of the existing literature on the effectiveness of PrEP in reducing HIV transmission rates. Additionally, we examine the obstacles related to PrEP implementation and uptake and put forward potential strategies to raise awareness and improve its use among populations at an increased risk of contracting HIV.

## 1. Introduction

Since the outbreak of the HIV/AIDS epidemic, approximately 84 million individuals have been infected with HIV, and over 40 million people have died due to AIDS-related illnesses [1]. In 2021, an estimated 38 million individuals worldwide were living with HIV, with approximately 0.7% of the global adult population aged 15–49 years being HIV-positive [1,2].

The burden of HIV/AIDS continues to vary considerably between countries and different population groups. According to the World Health Organization (WHO), the highest burden of HIV is found in Sub-Saharan Africa, where approximately 67% of all people living with HIV reside. In this region, women and girls are disproportionately affected, with young women (aged 15–24) being particularly vulnerable [2].

In developed countries, men who have sex with men (MSM) continue to be the most affected population group. In Europe, according to the ECDC 2021 report, 106,508 newly diagnosed HIV infections were reported in 46 of the 53 countries in the region. This corresponds to a crude rate of 12.0 newly diagnosed infections per 100,000 population in the region overall [2].

The proportion of all HIV diagnoses attributed to sex between MSM increased from 37% of cases with a known route of transmission in 2012 to 46% in 2021 in countries reporting consistently over the last decade [2,3]. However, the absolute number of HIV diagnoses reported among MSM in EU/EEA countries has declined since the end of 2015, even after adjusting for reporting delay [3]. The proportion of all HIV diagnoses attributed to heterosexually acquired infection in women remained stable between 2012 and 2021, ranging from 20% to 24%. The proportion of all HIV diagnoses attributed to heterosexually acquired HIV infection in men was also stable during this period, ranging from 19% to 21% [3].

Similar data are observed in Spain. Transmission in MSM is the most frequent in all age groups, accounting for 65.4% of new HIV diagnoses [4].

Persons who inject drugs (PWID) are another group with high HIV prevalence, particularly in regions where injection drug use is common. In many countries, harm-reduction programs, such as needle-exchange programs and opioid-substitution therapy, have been implemented to reduce the risk of HIV transmission among IDUs [1,2,3].

Other population groups with higher rates of HIV include sex workers, transgender individuals, and prisoners. It is important to note that these groups often face significant stigma and discrimination, which can hinder their access to HIV prevention, testing, and treatment services [5,6].

In order to manage the HIV epidemic, individualized strategies must be implemented to address the different groups at risk. A combination of prevention strategies, including condom use, pre-exposure prophylaxis (PrEP), and HIV testing and treatment, can help reduce the burden of HIV in these populations [1,3]. Additionally, addressing the underlying social determinants of health, such as poverty, discrimination, and gender inequality, is essential to achieving equitable HIV outcomes for all [7].

PrEP is one of the strategies applied to achieve the primary goal of eradicating the HIV pandemic [8]. It has emerged as an additional tool to combat HIV transmission and is recommended by UNAIDS as part of a combination prevention strategy [1,7]. PrEP involves giving antiretroviral therapy (ART) to people who do not have HIV to prevent infection. A daily regimen of TDF/FTC has been shown to be effective and safe in reducing the risk of transmission [9,10]. The international community has committed to ending the HIV/AIDS epidemic as a public health threat by 2030. This objective aligns with the ambitious goal of the 2030 Agenda for Sustainable Development, which was adopted by the United Nations General Assembly in September 2015 [8]. Although progress has been made in the fight against HIV/AIDS, much work remains to be conducted to achieve the goal of ending the epidemic by 2030. Focusing on addressing the specific challenges encountered by distinct population groups, including the deployment of PrEP and tackling the social determinants of health, is crucial in the ongoing battle against HIV/AIDS. In this article, we provide a concise overview of the primary components of this approach, emphasizing its effectiveness, safety, and future obstacles.

## 2. Brief History of PrEP: Development and Use in Different Populations

Pre-exposure prophylaxis (PrEP) has been crucial in HIV prevention since its approval by the U.S. Food and Drug Administration (FDA) in 2012 [11]. However, the concept of prophylaxis against HIV has been around since the 1980s, when the antiretroviral drug zidovudine (AZT) was used as a prophylactic agent in healthcare workers following accidental HIV exposure [12].

In the early 2000s, animal studies showed that other antiretroviral drugs, including tenofovir and emtricitabine, could also provide protection against HIV [13,14]. Published in 2010, the first human trial of PrEP was conducted among MSM in the United States [9]. This trial showed a 44% reduction in HIV incidence among those who received tenofovir as PrEP [9].

Subsequently, other clinical trials were conducted in different populations, including heterosexual couples in serodiscordant relationships, IDUs, and transgender women [9,10,15,16]. These trials demonstrated the efficacy of PrEP in reducing HIV acquisition, leading to the FDA’s approval of tenofovir/emtricitabine as PrEP in 2012 [17].

Since then, PrEP has been widely implemented as an HIV-prevention strategy in many countries around the world. However, there have been challenges in ensuring access to PrEP, particularly in low- and middle-income countries where HIV incidence is high and resources for healthcare are limited.

In recent years, there has been a growing interest in new forms of PrEP, including long-acting injectable formulations and on-demand or event-driven PrEP [16,18]. These new approaches may improve adherence to PrEP and reduce the burden of daily pill taking.

## 3. Understanding How PrEP Works to Prevent HIV Infection

PrEP medications work by targeting different stages of the HIV life cycle [1]. Two drugs that are commonly used for PrEP are tenofovir disoproxil fumarate (TDF) and emtricitabine (FTC) [17,19]. These drugs are taken orally, usually in combination, and are able to reach high concentrations in the tissues of the genitals and rectal mucosa, where the virus first enters the body during sexual intercourse [19,20].

Once PrEP medications are taken, they are absorbed into the bloodstream and distributed throughout the body. When HIV enters the body through sexual contact or injection drug use, the virus tries to enter CD4+ T cells, key cells of the immune system that are targeted and destroyed by HIV [21]. TDF and FTC operate by inhibiting the same enzyme: reverse transcriptase. This enzyme is necessary for HIV to replicate and establish a successful infection in CD4+ T cells [10,20,21,22]. By inhibiting these enzymes, TDF and FTC prevent the virus from making copies of its genetic material and integrating it into the DNA of host cells.

As a result, the virus is unable to establish a productive infection and is eventually cleared by the immune system. It is important to underline that PrEP medications need to be taken consistently and correctly in order to be effective. The optimal dosing schedule for PrEP is daily, but in certain populations, it can also be taken on-demand, before and after sexual activity, depending on the individual’s needs and preferences [16,17].

## 4. Effectiveness and Efficacy

The efficacy of oral PrEP as a prevention strategy against HIV was initially demonstrated in the iPREX study, where MSM subjects and transgender women in sexual relationships with men were randomized to receive either FTC/TDF or placebo [5]. The FTC/TDF group showed a 44% reduction in new HIV infections compared to the placebo group. Subsequent studies, such as the TDF2 study in Botswana and the Partners study in Kenya and Uganda, further confirmed the effectiveness of PrEP in reducing HIV transmission among heterosexual adults [23,24].

In the TDF2 study, FTC/TDF demonstrated 62.2% efficacy in preventing HIV transmission. Similarly, the Partners study, which involved serodiscordant heterosexual couples (where one *partner* is HIV-positive and the other partner is not), showed that both TDF and FTC/TDF regimens provided significant protection against HIV transmission, with efficacy rates of 67% and 75%, respectively [23,24].

Additional trials, such as PROUD (a real-life study) and IPERGAY (an on-demand study), focused on MSM populations and demonstrated the efficacy of daily or on-demand TDF/FTC in reducing the risk of HIV infection [20,25]. Adherence to the prescribed medication was found to be crucial to achieving high efficacy rates [5,20,25].

Furthermore, alternative strategies such as TAF (tenofovir alafenamide)/FTC have been explored, particularly for individuals with renal impairment or decreased bone density. The DISCOVER trial demonstrated that TAF/FTC is non-inferior to TDF/FTC, providing another viable option for HIV prevention [26]. However, it should be noted that the study did not include cisgender women in its study population; therefore, the results can only be applied to cisgender men and transgender women.

Other clinical trials included only cisgender heterosexual women (CAPRISA 004, FACTS-001 with Tenofovir, and ASPIRE, Ring Study Team with Dapivirine). In these studies, reduction in the risk of HIV infection was only demonstrated in cases with optimal adherence. [27,28,29].

In contrast to these studies, other studies have yielded conflicting results. The FEM-PrEP study involved 2120 HIV-negative women in Kenya, South Africa, and Tanzania who were randomly assigned to receive FTC/TDF or placebo. Out of the total participants, 33 women in the FTC/TDF group were infected (incidence of 4.7 infections per 100 person-years of follow-up [pyfu]), compared to 35 infections in the placebo group (5 infections per 100 pyfu), resulting in a non-significant protective hazard ratio (HR) of 0.94 (95% CI 0.59–1.52) [30]. Another study, namely the VOICE study (Vaginal and Oral Interventions to Control the Epidemic), conducted in South Africa, Zimbabwe, and Uganda included 5029 HIV-negative women who were randomized to different interventions: TDF alone; FTC/TDF; 1% TDF vaginal gel; and two placebo groups (one for oral treatment and one for vaginal gel). In total, 312 infections occurred with an incidence rate of 5.7 per 100 pyfu [31]. None of the three study arms showed significant protection compared to placebo. TDF administered as a single drug alone demonstrated a reduction of 49% in HIV incidence, while the combination of FTC/TDF showed a reduction of 4.4%. The vaginal gel exhibited an efficacy of 14.5%, although no statistical analysis was provided in this specific case [30,31]. Furthermore, there has been no evaluation conducted to date regarding the efficacy of the on-demand PrEP regimen in these individuals [16,17,19].

Another trial that considered the use of maraviroc (MVC), an HIV entry inhibitor, did not demonstrate efficacy [32].

One of the latest advancements in HIV prevention includes the introduction of the long-acting (LA) formulation of cabotegravir. The safety, tolerability, and acceptability of CAB-LA in HIV-uninfected individuals at low risk of infection were evaluated in the ECLAIR and HPTN077 studies [33,34], comparing it to a placebo. Both studies demonstrated that CAB-LA is a secure alternative to orally administered pre-exposure prophylaxis regimens.

Subsequently, in the HPTN 083 trial [35], the safety and efficacy of CAB-LA were compared to TDF-FTC for preventing HIV infection in MSM and transgender women engaging in sexual activities with men. The cabotegravir group exhibited a 66% lower risk of HIV infection compared to the TDF-FTC group, establishing the superiority of CAB-LA over TDF-FTC [35]. In the HPTN084 trial involving 3224 cisgender women, the CAB-LA group showed an 88% lower risk of infection compared to the TDF/FTC group for cisgender women [36].

Please refer to Table 1 for an overview of the studies discussed regarding PrEP.

## 5. Safety

Multiple clinical trials and real-world studies have demonstrated the safety of PrEP for HIV prevention. The most common side effects reported in clinical trials were gastrointestinal symptoms, such as nausea, vomiting, and diarrhea, which typically resolved within the first few weeks of treatment [37]. Other reported side effects included headache, fatigue, and dizziness, which were generally mild to moderate in severity [38].

There have been concerns that PrEP may increase the risk of kidney disease and bone loss. However, a meta-analysis of clinical trials and observational studies found no significant increase in the risk of kidney disease among PrEP users compared to placebo or no treatment. Similarly, several studies have reported no significant difference in bone mineral density between PrEP users and non-users [37,38].

Overall, the safety of PrEP has been well established through numerous clinical trials and real-world studies. Adverse effects are generally mild and transient, and serious adverse events are rare [10,27,38]. Ongoing monitoring and research are essential to ensure the safety of PrEP and to address any emerging concerns.

## 6. Concerns with PrEP: Resistance Mutations and STI Risks

Despite concerns about the potential emergence of resistance during PrEP, studies to date have not shown a significant increase in resistance development with the use of PrEP. In a review of over 10,000 people enrolled in various studies, only 18 (0.18%) of the 305 people who became infected during prophylaxis had resistance mutations. However, it is important to note that half of those who developed resistance mutations were subjects who had been included in the study during their primary infection [39]. Excluding this group, the rate of resistance development was only 0.09%. Similarly, the iPrEx study showed that only 2 out of 48 individuals who acquired HIV while taking TDF/FTC had resistance mutations [5], while the Partners PrEP study found that 5 out of 63 seroconverters (7.9%) in the active PrEP arms developed resistance [23]. These findings suggest that while the risk of resistance development is not negligible, it remains relatively low with the use of PrEP. Nevertheless, close monitoring of individuals initiating PrEP should be performed, particularly during the early phase of seroconversion when viral replication is high, to detect any potential development of resistance mutations.

Another possible controversy that arises in the implementation of PrEP is that its generalized use may lead to and increase the transmission of other sexually transmitted infections (STIs) apart from HIV.

In the last two decades, the incidence of sexually transmitted infections (STIs) has increased in our setting, particularly among MSM [40]. The rise in STI rates may be due to the widespread use of ART, which has led to a decreased perception of the severity of STIs, reduced condom use, and a preference for sexual partners with the same HIV status (serosorting) [4,10]. Studies have shown that MSM using PrEP for HIV prevention are at higher risk for bacterial STI acquisition than non-PrEP users [16]. However, it remains unclear whether PrEP use directly contributes to increased STI rates or whether PrEP users are simply more likely to acquire STIs due to other risk factors.

Recent studies have yielded confusing results on the impact of PrEP on STI incidence. For example, a study in Spain found no significant increase in STIs among PrEP users [41], while a French study found no significant decrease in STIs except during the COVID-19 confinement period. A large-scale study in Germany, on the other hand, found that nearly one in three MSM were diagnosed with at least one of the analyzed STIs, and that PrEP users had a higher prevalence of STIs compared to non-PrEP users [42]. It is unclear whether this is due to increased risk behavior or increased STI screening in PrEP programs. However, early detection and treatment of STIs through systematic screening in PrEP programs can help reduce their transmission [38].

Until now, the available evidence has suggested that PrEP is a safe and well-tolerated intervention for HIV prevention. However, it is important to note that PrEP is not 100% effective and should be used in combination with other prevention strategies, such as condoms and regular STI testing [2,3,4].

## 7. Future Challenges

### 7.1. Implementation

There are several caveats associated with PrEP implementation. One of the main challenges is the stigma associated with the use of antiretroviral drugs, which can deter some individuals from using PrEP [43]. Additionally, drug side effects, frequent relocation of beneficiaries, limited resources for routine screening and medication monitoring, and a limited number of qualified healthcare workers for PrEP distribution and administration pose significant barriers to PrEP implementation [43,44].

Another significant challenge is the low uptake of PrEP among populations at high risk of HIV infection. This matter can be attributed to a lack of knowledge and awareness about PrEP and its benefits, as well as accessibility issues in rural or remote areas [45].

Furthermore, the cost of PrEP can be a barrier to access, as it may be unaffordable for many individuals [46]. The lack of funding for research, development, and implementation of PrEP programs may also limit the capacity of healthcare providers to offer PrEP to those who need it.

### 7.2. Adherence

Adherence is a critical factor in the effectiveness of PrEP for HIV prevention. PrEP is most effective when taken consistently [5,23,25,37]. Studies have shown that PrEP adherence rates can vary widely depending on a variety of factors, including individual motivation, lifestyle factors, and healthcare support [5,6].

One important issue in PrEP adherence is education and awareness [5,26]. People who understand the benefits of PrEP and how to take it correctly are more likely to adhere to their medication schedule. Regular counseling and education sessions, along with effective communication between healthcare providers and patients, can help improve adherence rates [47].

Lifestyle factors can also play a role in adherence. Individuals who have busy schedules or travel frequently may find it difficult to maintain a consistent PrEP regimen [40]. Healthcare providers can work with patients to develop a personalized plan for PrEP adherence that takes into account their unique lifestyle factors [47]. In this regard, the dispensing of injectable medication may be a feasible solution.

The introduction of cabotegravir-PrEP is likely to increase adherence, improve implementation, and reduce AIDS-related deaths in addition to HIV incidence. A recent study published in *The Lancet* addresses these issues [48]. Focused on Sub-Saharan Africa, where HIV prevalence remains unacceptably high, the study simulated 1000 scenarios that reflected the variability and uncertainty of HIV epidemics in the region and compared the results with and without the introduction of cabotegravir-PrEP. The intention was to project the benefits and risks of introducing cabotegravir-PrEP to the region. The study found that the introduction of cabotegravir-PrEP could lead to a substantial increase in the use of PrEP, with approximately 2.6% of the adult population (and 46% of those with a current indication for PrEP) receiving PrEP compared to 1.5% without the introduction of cabotegravir-PrEP over a 20-year period. This is expected to result in a 29% lower HIV incidence (90% range in scenarios of 6 to 52%) compared to not introducing cabotegravir-PrEP [48]. However, it is likely to lead to an increase in resistance to integrase inhibitor drugs. Nonetheless, the study suggests that the introduction of cabotegravir-PrEP could reduce AIDS-related deaths, as well as HIV incidence.

The Levi Syndrome was recently reported as another potential downside of long-acting PrEP using Cabotegravir and rilpivirine [49]. This refers to the possible delay in diagnosing HIV infection through routine serological methods in individuals taking PrEP. The notable and sustained viral suppression observed with the use of long-acting Cabotegravir (CAB-LA) PrEP suggests that there might be limited establishment of the HIV reservoir during early stages of infection [49].

Another promising long-acting drug is being evaluated. Lenacapavir is an investigational first-in-class, long-acting HIV capsid inhibitor available as an oral formulation and as a subcutaneous injection administered every 6 months. It shows promising results as a long-acting PrEP option [50]. There are currently two planned phase III randomized trials to evaluate the efficacy and safety of lenacapavir for PrEP compared with a counterfactual placebo: PURPOSE 1 and PURPOSE 2 [51,52]. Both studies are open and actively recruiting participants.

### 7.3. Target Population

The target population for PrEP includes individuals who are at high risk of acquiring HIV, such as men who have sex with men, transgender individuals, and persons who inject drugs. However, there are also subgroups within these populations that may face even greater risks and may therefore benefit from PrEP [5,6,43].

Sex workers are a key population in the global HIV epidemic. In 2019, the Joint United Nations Programme on HIV/AIDS estimated a mean HIV prevalence of 36% among sex workers [53]. They face high levels of stigma and criminalization almost everywhere. Modelling studies indicate that decriminalizing sex work could lead to a 46% reduction in new HIV infections in sex workers over 10 years, while eliminating sexual violence against sex workers could lead to a 20% reduction in new HIV infections [53].

Studies have shown that PrEP can be highly effective in reducing HIV incidence among sex workers. Daily oral PrEP reduced the risk of HIV infection among female sex workers by 75% [54]. In addition, studies have shown that PrEP can be highly effective in reducing HIV transmission among transgender women who engage in sex work.

Research has shown that sex workers are interested in using PrEP but face a range of barriers to accessing and using it. These barriers include lack of information, stigma and discrimination, criminalization, and concerns about confidentiality and privacy [5,54].

To address these barriers, a number of initiatives have been implemented to increase access to PrEP. For example, community-led programs have been developed to provide education and support for sex workers around PrEP, as well as to address issues such as stigma and discrimination. In addition, some countries have developed national PrEP programs that specifically target sex workers [55].

Transgender individuals also face disproportionate rates of HIV acquisition. According to the Centers for Disease Control and Prevention (CDC), transgender women have an HIV prevalence rate of 42% compared to 0.3% in the general population in the United States [56]. Discrimination, violence, and lack of access to healthcare and social services are some of the factors that contribute to this heightened risk. Transgender individuals may experience stigma and discrimination in healthcare settings, which can discourage them from seeking preventive care and demanding PrEP.

Research has shown that PrEP is highly effective in reducing HIV incidence among transgender individuals [5]. A recent study found that injectable cabotegravir was highly effective in preventing HIV transmission among cisgender men and transgender women who have sex with men [35].

However, despite the effectiveness of PrEP, transgender individuals may face multiple drawbacks, such as lack of awareness, cost, insurance coverage, and stigma. It is essential to address these barriers and ensure equitable access to PrEP for transgender individuals to reduce the disparities in HIV acquisition.

## 8. Conclusions

PrEP has emerged as a highly effective tool for preventing HIV transmission, but access to it remains a challenge in many parts of the world. Ongoing research and efforts are needed to optimize PrEP implementation and improve access for those who need it the most.

One way to achieve this is by identifying barriers that may hinder the implementation and continuity of PrEP. Studies have highlighted the importance of reducing stigma and improving confidentiality to increase PrEP uptake among key populations.

Moreover, new PrEP formulations, such as injectable long-acting PrEP, offer increased convenience and adherence for certain populations. Injectable cabotegravir is demonstrating efficacy in preventing HIV transmission among cisgender men and transgender women who have sex with men. This new formulation could be particularly beneficial for individuals who struggle with adherence to daily oral PrEP.

Overall, ongoing research and efforts to address access barriers and improve adherence to PrEP are critical for achieving the goal of virtual elimination of HIV in the coming years. PrEP is a powerful asset in the fight against HIV, and it is essential that we continue to work towards making it more accessible and effective for all those who could benefit from it.

## Figures and Tables

**Table 1:** Summary of clinical trials.

Clinical Trial	Participants	Success Rate
iPREX Study	MSM subjects and transgender women in sexual relationships with men	44% reduction in new HIV infections compared to placebo [5]
TDF2 Study	High-risk individuals in Botswana	62.2% efficacy in preventing HIV infections [23]
Partners Study	Serodiscordant heterosexual couples in Kenya and Uganda	TDF: 67% efficacy; FTC/TDF: 75% efficacy in preventing HIV transmission [24]
PROUD Study	MSM populations	Daily TDF/FTC: Effective in reducing the risk of HIV infection [20]
IPERGAY Study	MSM populations	On-demand TDF/FTC: Effective in reducing the risk of HIV infection [25]
DISCOVER Trial	Cisgender men and transgender women	TAF/FTC: Non-inferior to TDF/FTC, providing another option for HIV prevention [26]
CAPRISA 004	Cisgender heterosexual women	Reduction in the risk of HIV infection demonstrated with optimal adherence [27]
FACTS-001 with Tenofovir	Cisgender heterosexual women	Reduction in the risk of HIV infection demonstrated with optimal adherence [28]
ASPIRE	Ring Study Team with Dapivirine	Cisgender heterosexual women [29]
FEM-Prep Study	HIV-negative women in Kenya, South Africa, and Tanzania	No statistically significant reduction in HIV infections observed [30]
Voice Study	HIV-negative women in South Africa, Zimbabwe, and Uganda	No significant reduction in HIV infections observed [31]
Maraviroc (MVC) Trial	Various populations	Did not demonstrate efficacy [32]
éclair	HIV-uninfected individuals at low risk of infection	Safety of CAB-LA [33]
HPTN077	HIV-uninfected individuals at low risk of infection	Safety of CAB-LA [34]
HPTN 083	MSM subjects and transgender women in sexual relationships with men	CAB-LA: 66% lower risk of HIV infection compared to the TDF-FTC group [35].
HPTN084	Cisgender heterosexual women	CAB-LA: 88% lower risk of infection compared to the TDF/FTC group [36]

## Data Availability

The availability of the data supporting the conclusions of the article is public. It is reflected in the references.
